# Witnessing workplace bullying — protocol for a systematic review and meta-analysis of individual health and well-being outcomes

**DOI:** 10.1186/s13643-023-02288-0

**Published:** 2023-07-10

**Authors:** Morten Birkeland Nielsen, Michael Rosander, Ståle Valvatne Einarsen

**Affiliations:** 1 National Institute of Occupational Health, PB 5330 Majorstuen, 0304 Oslo, Norway; 2 Department of Psychosocial Science, University of Bergen, Postboks 7807, 5020 Bergen, Norway; 3 Department of Behavioural Sciences and Learning, Linköping University, 581 83 Linköping, Sweden

**Keywords:** Harassment, Aggression, Witnessing, Observers, Literature review

## Abstract

**Background:**

Most research on workplace bullying has examined the impact of the mistreatment on those exposed. Although bullying also is assumed to have significant ripple effects on bystanders, the empirical evidence for this line of research is highly fragmented and inconclusive. The overarching aim of this planned systematic review and meta-analysis is therefore to determine whether witnessing bullying of others at the workplace is associated with health problems and lower well-being among the observers. To achieve this aim, the review includes an assessment of which theoretical frameworks and methodological designs used in research so far and shed light on which confounders, mediators, and moderators that have been accounted for.

**Methods:**

A systematic review and meta-analysis will be conducted. Electronic databases will be searched using pre-defined search terms to identify relevant studies. Eligible studies should report empirical findings on any individual outcome variable assessed among witnesses to workplace harassment and bullying or any overlapping concept. Primary observational studies with cross-sectional or prospective research design, case–control studies, and studies with experimental designs will be included. Qualitative interviews and case studies will be excluded. The methodological quality of the included studies will be assessed with a previously established checklist for studies on workplace bullying. The quality of evidence for an association between witnessing bullying and potential outcomes will be evaluated in accordance with the GRADE system. A random effects meta-analysis will be conducted with the Comprehensive Meta-Analysis software, version 3.

**Discussion:**

We expect that findings on outcomes of bystanding to workplace bullying will provide practitioners with an understanding of the effects workplace bullying may have also on non-targets and the workplace as a whole. Such information is important regarding the development and implementation of effective measures and interventions against bullying. In addition, the review will increase our understanding of existing research gaps and enable us to make recommendations to address them. Our work aligns with the sustainable development agenda to protect workers and reduce inequalities at the workplace.

**Systematic review registration:**

PROSPERO 342006.

Workplace bullying refers to a systematic form of harassment where an employee persistently and over a period of time is exposed to negative actions from others at the workplace (e.g. colleague, superiors, or subordinates) and where the employee finds it difficult to defend themself against these actions due to a perceived power imbalance between target and perpetrator [1, 2]. In a workplace context, bullying mainly involves repeated exposure to verbal hostility, being made the laughing stock of the department, having one’s work situation obstructed or being given unreasonable tasks, or being socially excluded [3]. Our empirical knowledge and understanding of workplace bullying have grown extensively over the last three decades [4], and the evidence on prevalence rates, costs, antecedents, outcomes, and mechanisms has been summarized in multiple systematic reviews and meta-analyses [2]. Taken together, this synthesized evidence shows that bullying is a frequent stressor with a global prevalence rate of about 15% [5], and that bullying is associated with a range of outcomes for those exposed, including mental health problems [6, 7], somatic complaints [8], sleep problems [9], and suicidal ideation [10], as well as with reduced job satisfaction [11] and work ability [12].

Considering that the workplace is highly important regarding the financial situation and the personal identity and well-being of all employees, it is not surprising that being a target of prolonged harassment and social exclusion at work has severe and detrimental consequences for the health and well-being of those directly exposed. However, as bullying at the workplace occurs within a social context, the phenomenon has also been suggested to have negative ripple effects for others in the work environment and especially for bystanders, that is those who witnesses the bullying while not being directly targeted themselves [13, 14]. Specifically, building on a “victim by proxy” hypothesis, it is assumed that observing the bullying of others can be perceived as threatening as it may indicate how other employees at the workplace can be treated. If one employee is treated badly, one may also risk similar negative treatment in the future as a bystander [15]. Building on the stressor-strain appraisal theory [16] and conservation of resources theory [17], Sprigg and colleagues [18] argued that “the effects of witnessing bullying on employees’ well-being emanate from a two-stage appraisal process in which employees appraise the situation or event they have witnessed and whether it poses a threat to them (primary appraisal), and then assess whether they are able to deal with what they have witnessed (secondary appraisal)”. They further highlighted the importance of moderators in this process by claiming that the availability of both personal and contextual resources is expected to therefore determine the magnitude of the outcomes associated with witnessing workplace bullying at the primary appraisal stage and their ability to cope at the secondary appraisal stage.

Despite these theoretical reasons for expecting that witnessing the bullying of others to be a risk factor for the health and well-being of the bystander, there are important knowledge gaps regarding bystanders to bullying [2, 13]. Furthermore, while Niven and colleagues (2020) provided a thorough summary of research on bystanders in book chapter format, a full systematic literature review and meta-analysis has to this date not been conducted. To add to our knowledge about the potential impact of bullying on bystanders, this planned systematic review and meta-analytic study will therefore provide a synthesis of all available primary studies on individual health and well-being outcomes following witnessing bullying of others at the workplace. The overarching objective of the review will be *to determine whether witnessing bullying of others at the workplace is associated with health problems and lower well-being among the observers*. To achieve this main objective, it is crucial to know the nature and content of the existing research base, including the outcomes examined, the theoretical rationales, and methodological approaches. The review will therefore be guided by five research questions:

A) Which health and well-being outcomes that have been examined in research on bystanders so far (and thereby also answer which outcomes that have not been investigated)?
B) What is the magnitude of the associations between witnessing bullying and the health and well-being outcomes?
C) Which theoretical frameworks have been used to explain the impact of bystanding on outcomes?
D) What are the methodological designs that have been used in research on bystanders?
E) Which confounders, mediators, and moderators have been accounted for in research to this date?

Hence, we will, in a systematic manner, describe the nature of current research on bystanders of bullying and provide analyses of its strengths and limitations to date. In the meta-analytic part of the review, we will determine the magnitude of the association between observing workplace bullying as a bystander and outcomes.

## Methods

This protocol has been written based on the PRISMA-P (Preferred Reporting Items for Systematic Review and Meta-Analysis Protocols) guidelines and the MOOSE Guidelines for Meta-Analyses and Systematic Reviews of Observational Studies [19, 20]. A preliminary search of PROSPERO, MEDLINE via PubMed, CINAHL, the *JBI Database of Systematic Reviews and Implementation Reports*, and the Cochrane Database of Systematic Reviews did not reveal any currently ongoing or completed systematic reviews related to health and well-being outcomes among bystanders to workplace bullying.

### Data sources search terms and search strategy

This literature review and meta-analysis will be based on systematic searches in the following databases: MEDLINE/PubMed, ProQuest, Web of Science, Taylor & Francis Online Journals, PsychINFO, and Wiley Online Library. Additional searches will be performed in Scopus and Google Scholar. All search terms are included in Table 1. The systematic searches will be conducted by combining every possible combination of the three categories of keywords. In line with previous systematic reviews and meta-analyses on workplace bullying [9, 11], the searches will not be limited by historical time constraints. The systematic procedure substantiates that the literature search comprises all published studies on bystanders of workplace bullying. Consequently, no specific search terms of health and well-being outcome variables will be included as that is one of the outcomes of the planned review. Information about the outcomes of witnessing bullying will be extracted manually during the screening of studies. Through being general and wide, the search strategy is considered to reduce the risk of selection and detection bias.

**Table 1:** Overview of search terms in this review

Group 1	Group 2	Group 3
Work*	Bullying	Witness*
Job	Mobbing	Observ*
Occupation*	Victimization	Bystand*
Employee	Emotional abuse	Third part*
Organization*	Incivility	Whistleblow*
	Psychological aggression	Co-worker
	Mistreatment	Colleague
	Ostracism	
	Exclusion	
	Undermining	
	Harassment	
	Cyberbullying	
	Cyberharassment	

^*indicates Medical Subject Headings terms; MeSH^

A professional librarian will conduct the search. The primary investigator will oversee the search strategy. The search results will be exported to Covidence, a web-based screening and data extraction tool for authors conducting systematic reviews. After duplicates are removed, studies extracted from the literature search will be screened by two reviewers in order to determine eligible studies. Inclusion and exclusion criteria are described below. Any differences in opinions will be resolved through discussion until a consensus is reached. A third reviewer may be consulted if necessary. This process ensures that bias is minimized when deciding whether to include or exclude a given study. The two reviewers will independently conduct the data extraction from each study using a pre-defined data extraction sheet. Following the description by Lipsey and Wilson [21], the coding form will assess information about witnessing bullying, outcomes, demographic characteristics of participants (age, gender, job type, employment status, educational level, etc.), study characteristics (country of origin, sample size, effect sizes, response rate, year study published, sampling method, measurement inventories etc.), and other relevant variables (health indicators, other exposures). Reference lists of included full-text articles will be manually inspected to detect any potentially eligible studies.

### Primary outcome variables of interest

This review will be restricted to outcome variables related to health and well-being of bystanders to bullying at the individual level. According to Danna and Griffin [22], “Well-being is viewed as comprising the various life/non-work satisfactions enjoyed by individuals, work/job-related satisfactions, and general health. Health, in turn, is seen as being a sub-component of well-being and comprises the combination of such mental/psychological indicators as affect, frustration, and anxiety and such physical/physiological indicators as blood pressure, heart condition, and general physical health” (p. 359). Hence, this planned review on bystanding to bullying will include outcome variables related to mental health problems (e.g. anxiety, depression, emotional exhaustion, burnout), somatic complaints (e.g. headache, stomachache, musculoskeletal complaints), sleep, sickness absence, life satisfaction, job satisfaction, turnover intent, and organizational commitment. Other related outcome variables may be added if identified through the review process.

### Inclusion and exclusion criteria

Eligible studies should report empirical findings on any individual health and well-being outcome variable assessed among witnesses to workplace harassment or bullying (or any overlapping concept). Primary observational studies with cross-sectional or prospective research design, case–control studies, and studies with experimental designs will be included. Cross-sectional data will be used to determine the magnitude of the association between witnessing bullying and the assessed outcome variables, whereas prospective data will be used to determine directions of associations. As the strength of associations based on prospective data is likely to be a function of the utilized time-lag between measurement points [23], it is important to also include cross-sectional data [24]. Qualitative interview studies, single case studies, and series of single case studies will not be included in the review or meta-analysis.

For the meta-analytic part of the study, included studies are required to provide the zero-order associations between witnessing bullying and outcomes or provide sufficient information for these associations (effect sizes) to be calculated. Studies lacking this information or reported effect sizes that could not be transformed into correlations will be excluded from the meta-analysis. To avoid double-counting data, the sample in a given study should not have been used in a previous study of those included in the review. In cases with overlap, we will use data from the largest sample. The review will be limited to articles published in peer-reviewed journals in English, German, French, or the Scandinavian languages (Danish, Norwegian, and Swedish). Hence, this will be a review of published peer review studies only. Accordingly, data based on conference abstracts, dissertations, and grey literature (e.g. reports) will not be included. As a first step, relevant articles will be considered based on their title and abstract. At the second step, full-text versions of selected papers will be examined and assessed regarding effect sizes and methodological quality.

### Participants

The study population will be adults (18 years or older) with a current or previous employment in a full- or part-time position. No restrictions will be placed on participants’ gender, ethnicity, or other demographic characteristics. A minimum of two studies is considered sufficient to perform a meta-analysis [25].

### Assessment of methodological quality (risk of bias)

As displayed in Table 2, the methodological quality of the included studies will be assessed with an adapted version of a previously established checklist for research on workplace bullying comprising 14 items related to sampling, representativeness, measurement issues, and confounders [9, 12]. This checklist comprises selected and adapted items from the Risk-of-Bias Assessment Tool for Nonrandomized Studies [26] and the Quality Assessment Tool [27]. The quality of the reviewed studies will be scored on a scale from 0 (lowest possible quality) to 14 (highest possible quality). Kappa will be calculated to quantify the level of inter-rater agreement.

**Table 2:** Checklist for the assessment of the methodological quality of the reviewed studies

**Part 1 Sampling and representativeness**		Points
1. Sampling method		
A	Non-probability sampling (including purposive, quota, convenience and snowball sampling)	0
B	Probability sampling (including simple random, systematic, stratified, cluster, two-stage and multistage sampling)	1
2. Was the response rate reported?		
A	Not reported	0
B	Response rate below 50%	0
C	Response rate at 50% or above	1
3. Are the individuals selected to participate in the study likely to be representative of the target population?		
A	No	0
B	Yes	1
4. Selection bias: Is there a risk of selection bias caused by the inadequate selection of participants		
A	High risk	0
B	Low risk	1
5. Is the sample size adequate for establishing relationships (assumption of statistical power)		
A	No	0
B	Yes	1
**Part 2 Measurement and confounders**		
6. How was workplace bullying measured?		
A	Self-labeling without definition of the bullying concept	0
B	Self-labeling with a definition of the bullying concept	1
C	Behavioural checklist (e.g. NAQ, LIPT)	1
7. How was bystanding assessed?		
A	Single-item question	0
B	Behavioural checklist	1
8. Performance bias: Is there a risk of performance bias caused by the inadequate measurement of exposure		
A	High risk	0
B	Low risk	1
9. Are the statistical methods appropriate for the study design?		
A	No/cannot tell	0
B	Yes	1
10. Were meaningful demographic covariates included?		
A	No	0
B	Yes	1
11. Were other work factors adjusted for?		
A	No	0
B	Yes	1
12. Is the study design cross-sectional or prospective (with time lag)?		
A	Cross-sectional	0
B	Prospective	1
13. Was previous the outcome variables adjusted for in prospective analyses?		
A	No	0
B	Yes	1
14. Confounder bias: Is there a risk of bias caused by the inadequate confirmation and consideration of confounding variable		
A	High risk	0
B	Low risk	1

The quality of evidence for an association between witnessing bullying and outcomes will be evaluated in accordance with the GRADE system [28]. This system grades quality of evidence at four levels: high (4), moderate (3), low (2), and very low (1). For high evidence, the requirements are a randomized, doubled-blinded study design with no selection biases. For observational studies, moderate evidence, that is, exceptionally strong evidence from unbiased studies, is considered the strongest possible level of proof for an association.

### Meta-analytic approach

The meta-analysis will be conducted with the Comprehensive Meta-Analysis (version 3) software developed by Biostat [29]. Odds ratio (OR) with 95% confidence intervals (95% CI) will be reported as an overall synthesized measure of effect size. The mean of the combined effect sizes will be calculated in studies where several effect sizes were reported from the same sample (e.g. models with different control variables). An overall estimate will be calculated for studies with overlapping samples. In studies reporting effect sizes from independent subgroups (e.g. moderators), each subgroup will be included as a unique sample in the meta-analysis. Moderation analyses will also be used to compare associations from cross-sectional and prospective data. In contrast to some other meta-analytic methods, such as the Hunter and Schmidt approach [30], which weights studies by sample size, the Comprehensive Meta-Analysis programme weights studies by inverse variance. Inverse-variance weighting is a method of aggregating two or more random variables where each random variable is weighted in inverse proportion to its variance to minimize the variance of the weighted average. The inverse variance is roughly proportional to sample size, but is a more nuanced measure, and serves to minimize the variance of the combined effect [31].

As the individual studies included cannot be expected to come from the same population of studies, pooled mean effect size will be calculated using the random effects model. Such effects models are thus recommended when accumulating data from a series of studies where the effect size is assumed to vary from one study to the next and where it is unlikely that studies are functionally equivalent [31]. Random effect models allow statistical inferences to be made to a population of studies beyond those included in the meta-analysis [32]. The Q_within_ statistic will be used to assess the heterogeneity of studies. A significant Q_within_ value rejects the null hypothesis of homogeneity. A *I*^2^ statistic will be computed as an indicator of heterogeneity in terms of percentages. Increasing values show increasing heterogeneity, with values of 0% indicating no heterogeneity, 50% indicating moderate heterogeneity, and 75% indicating high heterogeneity, respectively [33]. The “one-study-removed” procedure will be used as a sensitivity analysis to determine whether the overall estimates between witnessing bullying and potential outcomes are influenced by outlier studies. Using this approach, effect sizes that fall outside the 95th confidence interval of the average effect size will be considered as outliers. Four indicators of publication bias are to be examined: funnel plot, Rosenthal’s fail-safe N, Duval and Tweedie’s trim and fill procedure and Egger’s regression intercept [34].

The inclusion of a meta-analysis in this review will depend on two requirements:

- First, whether we can identify enough primary studies that report effect sizes on the same outcomes. It has previously been proposed that a quantitative synthesis needs at least two studies [35], but more studies are likely to provide more unbiased results.
- Second, whether the outcomes are sufficiently similar to warrant their combination into an overall result. If studies are too heterogenous or based on many different tools and measures, it may not be possible to perform a meta-analysis.

## Discussion

This planned review and meta-analysis will systematically explore the evidence available on health and well-being outcomes of being a witness to workplace bullying. By gathering and summarizing information about magnitude of effect sizes, theoretical models employed, methodological designs prevailing the field, and the mediating and moderating factors studied in relation to how witnessing bullying of others influence bystanders, the findings from this study will provide directions for future research and provide practitioners with an understanding of the effects workplace bullying may have also on non-targets and the workplace as a whole. This knowledge can then be used to develop stronger countermeasures and interventions.

### Limitations

As data will be extracted using full-text articles only, and excluding data from grey literature, this review will build on published studies and doctoral dissertations exclusively, whereas unpublished studies and non-peer-reviewed literature (e.g. reports) are to be excluded. Although it has been suggested that researchers should aim at including unpublished literature in meta-analyses and systematic reviews, the inclusion of data from unpublished studies can itself introduce bias [36]. First, the unpublished studies that can be located are likely to be an unrepresentative sample of all unpublished studies. For instance, the identification of unpublished studies may depend on the willingness of investigators of unpublished studies to provide data. This may again depend upon the findings of the study, with more favourable results being provided more readily shared [36]. Secondly, unpublished studies may be of lower methodological quality than published studies. In a study of 60 meta-analyses that included published and unpublished studies, it was found that unpublished studies were less likely to conceal intervention allocation adequately and to blind outcome assessments [37]. As the planned review will be based on a comprehensive literature search of studies published in peer-reviewed journals, the included studies should be representative for the published literature on bystanders to workplace bullying. In addition, the peer review process should, at least in theory, ensure some degree of scientific quality of the included studies. The robustness of the findings will also be indicated by publication bias analyses. However, even though the papers are published in peer-reviewed journals, there is a potential risk of bias in the peer review process that allows lower quality studies to be published. For this reason, quality assessment of each study will be performed, and the robustness of the review will be further provided by this assessment.

It is likely that most associations reported in primary studies will be based on self-report data based on from self-administered questionnaires. This kind of data is prone to be influenced by common method bias as well as response set bias such as expectations, previous experiences, or health status. This may cause both non-differential and differential misclassification, resulting in under- and overestimations of effects [38]. The meta-analysis will include studies with cross-sectional designs, and the aggregated effect sizes from these studies will therefore not allow for conclusions about cause-and-effect relationship between the included variables. However, cross-sectional studies can be an important starting point for establishing connections among variables that can serve as the basis for further understanding and theorizing [24]. In the context of bystanding to workplace bullying, cross-sectional findings represent the first step in figuring out whether witnessing bullying of others might be a cause of health problems and low well-being. In addition, to add to the understanding of causal relations, separate analyses will be conducted for studies based on time-lagged data to determine direction of associations over time in the planned review.

### Ethics and dissemination

Ethical approval is not required for this systematic review and meta-analysis as only a secondary analysis of data already available in scientific databases will be conducted. The results of this review will be submitted for peer-reviewed publication and will be presented at relevant conferences.

### Review status

This review is expected to be complete by August, 2023.

## Data Availability

Not applicable.
